# Genetic variants associated with neurodegenerative Alzheimer disease in natural models

**DOI:** 10.1186/s40659-016-0072-9

**Published:** 2016-02-26

**Authors:** Claudia Salazar, Gonzalo Valdivia, Álvaro O. Ardiles, John Ewer, Adrián G. Palacios

**Affiliations:** Facultad de Ciencia, Centro Interdisciplinario de Neurociencia de Valparaíso, Universidad de Valparaíso, Valparaíso, Chile; Centro Interdisciplinario de Neurociencia de Valparaíso, Universidad de Valparaíso, Pasaje Harrington 287, Playa Ancha, 2360102 Valparaíso, Chile

**Keywords:** Degus, Genome, APP, APOE

## Abstract

The use of transgenic models for the study of neurodegenerative diseases has made valuable contributions to the field. However, some important limitations, including protein overexpression and general systemic compensation for the missing genes, has caused researchers to seek natural models that show the main biomarkers of neurodegenerative diseases during aging. Here we review some of these models—most of them rodents, focusing especially on the genetic variations in biomarkers for Alzheimer diseases, in order to explain their relationships with variants associated with the occurrence of the disease in humans.

## Background

A valuably strategy for the study of neurodegenerative diseases like Alzheimer’s disease (AD), has been the use of transgenic mice bearing a particular human allele, to evaluate its pathogenic potential [43]. Unfortunately, most transgenic models don’t recapitulate the full spectrum of a particular disease and require protein overexpression. Although new knock-in mouse models promise to show a more realistic and faithful progress of human diseases [85], nevertheless the short life of mice still prevents an accurate association between age and sporadic diseases. Therefore, a promising alternative approach is the search for non-transgenic models (NTM), in which the main hallmarks of a pathological phenotype appear naturally during aging [15]. More recently, the extraordinary advent and growth in genomic information has lead to the availability of complete genomes from a large number of different species. The latter offer a unique opportunity to investigate the involvement of particular genes in different diseases, in NTM. So it is possible today to ask, What might be the genetic basis of a neuropathology? What would be the importance of inherited or risk genes for the start and/or progress of AD pathology?

Here we review NTM of neurodegeneration and use published genomes to compare the sequences of specific gene variants in relation to idiopathic or sporadic form of AD (SAD). The latter in order to identify in NTM of AD gene sequences (tau, APOE, APP, PSEN, Aβ) corresponding to gene variants for causing AD in human.

### Genes implicated in familial Alzheimer´s disease

**Fig. 1:**
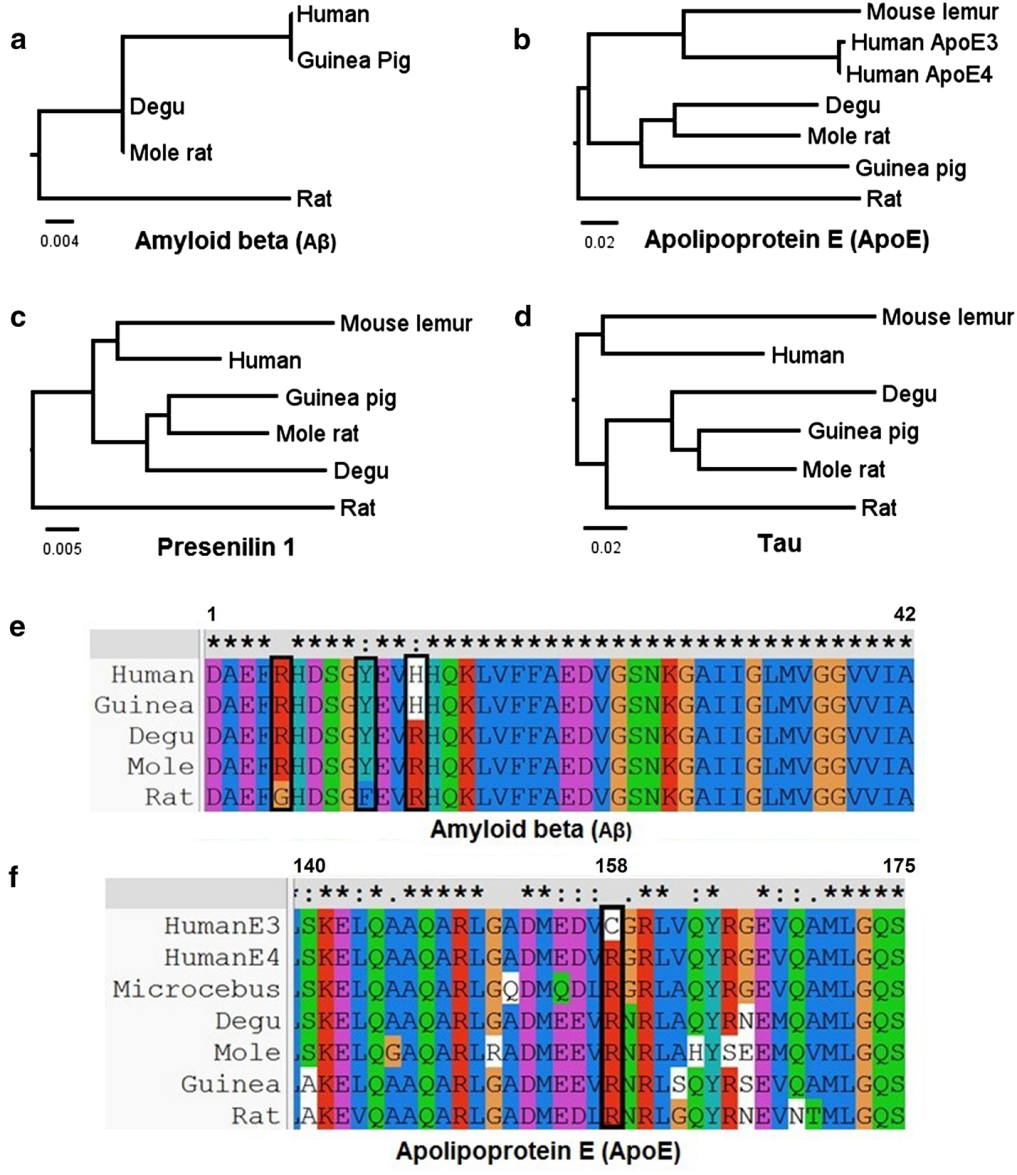
Phylogenetic analysis of 4 proteins involved in Alzheimer disease. **a** Phylogenetic tree of Aβ. The tree shows that the sequence for the degu is identical to that of the mole rat and is more similar to that of human and guinea pig than it is to that of the rat. **b** Phylogenetic tree of ApoE, showing that degu ApoE is more similar to the human protein than it is to rat ApoE. **c** Phylogenetic tree of Presenilin 1. The tree shows that the sequence for the degu is grouped with that of the mole rat and the guinea pig and is separate from the human and mouse lemur grouping. As occurs for Aβ and ApoE proteins, the Presenilin 1 sequence for the rat is outside of both groupings. **d** Phylogenetic tree of tau. In this case, the sequence for the rat is grouped with that of other rodents. **e** Multiple alignment of Aβ shows the three aminoacids (*boxed*) that differ between the rat and the other species; by contrast there is only one difference between degu and human APP sequence (H684R). **f** Multiple alignment of ApoE. There is a single difference between human ApoE3 and ApoE4 alleles (C112R, *boxed*); interestingly, this ApoE4 variant is the one that is present in all the other species. Aβ mouse lemur sequence is absent in the phylogram showed in **a**, because it was not available at the time of analysis

AD in its familial (early onset) or sporadic (late onset) form is characterized by the occurrence of a series of critical biomarkers. Among these the main indicators of neural degeneration, including synaptic failure and cognitive decline [88] are the accumulation of phosphorylated tau protein, which form neurofibrillary tangles (NFT), and the overexpression of amyloid precursor protein (APP), which leads to the accumulation of Amiloid-β (Aβ) peptide in senile plaques,

APP is an integral membrane protein present in the brain [50] and has been related to diverse functions including cell adhesion, growth factor and signaling associated with synaptogenesis and synaptic plasticity [98]. The proteolytic processing of APP releases potentially neurotoxic species, e.g., the Aβ peptide, which is considered one of the key pathogenic events in AD. The deregulation of both APP and Aβ has been linked to the hereditary or familial form of AD (FAD). For example, the Aβ peptide is present in meningovascular brain deposits in AD and in Down syndrome patients [46, 47], and is also a main component of senile plaques [49, 71, 89]. The cloning of the APP gene [58] showed that more than 20 mutations are associated to FAD. APP belongs to a conserved superfamily, which in mammals includes APP and the APP-like proteins, APLP1 and APLP2 [25]. APP isoforms 770, 751, and 695 residues in length, are produced by alternative splicing of exons 7 and 8 [90], the latter being the most abundant form in neurons [51]. A comparative analysis of APP revealed the existence of an important number of conserved amino acids [57]; Fig. 1). The Aβ region differs by 3 amino acids in rodents vs. humans (R5G, Y10F and H13R). In fact, the presence of these residues affects APP processing through its aminoacid oxidation induced by free radical generation systems and the subsequent generation of cross-linking protein interactions [37]. Interestingly, it has been suggested that the absence of these residues in Aβ rodent makes it less prone to forming amyloid aggregates [41, 77]). These differences between species could also provide protection against β-secretase processing [28]. This may also be the reason why the transgenic mouse model of AD requires human APP to be over-expressed in order to cause the AD pathogenesis, a feature that limits the utility of such models [32, 45].

Other proteins involved in FAD are presenilins [87]. Mutations in the *presenilin 1* gene (PSEN1) are present in certain families with clinical early onset AD [93], whereas mutations in the *presenilin 2* gene (PSEN2) appear to lead to AD at a more advanced age [23, 64, 83]. Presenilins are catalytic proteins of γ-secretase complexes responsible for producing Aβ from APP [52]; thus, mutations in PSEN1 and PSEN2 affect the generation of Aβ peptide [13, 22, 27, 35, 40, 95]. To date, over 180 mutations in PSEN1 and 15 mutations in PSEN2 have been reported with potential pathogenicity related to AD. Remarkably, the incorporation of transgenes bearing missense mutations in presenilins in APP transgenic mice seems to accelerate the processing and production of Aβ [35, 53].

The search in human cerebrospinal fluid for proteins that could bind Aβ peptides led to the identification of apolipoprotein E (ApoE), which was classified as a genetic risk factor in SAD [97]. There are three alleles of ApoE: ApoE2, ApoE3, and ApoE4. The age of onset of AD decreases with increasing copies of the ApoE4 allele [24, 84] such that, for example, homozygosity for this allele increases the risk of AD 12-fold [24, 39]. Nevertheless, the inheritance of ApoE4 is not sufficient for the development of AD. Interestingly, the deletion of the two copies of mouse ApoE in APP transgenic mice causes a reduction in amyloid accumulation [4] and APP mice expressing human ApoE3 or ApoE4 develop less Aβ deposits than do animals without ApoE [54].

Another key factor for AD is the microtubule binding protein, tau, for which its hyperphosphorylated form is a component of neurofibrillary tangles (NFT) [60, 73, 102]. NFT are present in both familiar and sporadic forms of AD; however, to date specific mutations in the MAPT gene haven’t been associated with AD, suggesting that the tau pathology occurs downstream of the detrimental cascades caused by Aβ. Tau depositions are also associated with a number of other tauopathies, including frontotemporal dementia (FTD), Pick disease, and corticobasal degeneration [91]. Several mutations have been identified in tauopathies [100], which have then been used to generate transgenic mice for the study of this class of diseases [6]. Furthermore, a transgenic tau mouse related to FTD and bearing mutations in APP and PSEN1, have provided evidence that amyloid deposition develops prior to tangle pathologies [74] and that memory deficits are related to increases in Aβ [82].

### Natural models of AD

During the last decades, and prompted by an understanding of the genetic basis of AD, several transgenic mice models have been developed that attempt to recreate sequence variants associated with FAD [36]. Moreover, newer knock-in mice models show a more realistic development of AD avoiding the overexpression of APP and of APP fragments such as Aβ, by showing a  more precise pattern of expression [21, 65, 85]. However, despite these advantages the limited lifespan of mice precludes the appropriate analysis of these models in the context of normal aging. Nevertheless, the advent of modern genetics and bioinformatics has led to a more comprehensive characterization of the genomes of animal models and has facilitated the search of NTM for which aging is associated with neurodegeneration [68]. Below, we describe some NTM that are relevant for the study of AD (Table 1).

**Table 1:** Natural animal models of AD

Model	Lifespan	Neuropathology	Reference
Naked mole-rat	28–32 years	Decreased Tau phosphorylation and amyloid-β accumulation starting at 3 months of age	[38, 76]
Mouse lemur	10–15 years	Senile plaques and neurofibrillary tangles present in cortex at 9 years of age, and hippocampus at 10 years. Procedural memory is normal, but executive function is altered at 7–11 years	[79, 94]
Octodon degus	10–12 years	Amyloid-β oligomers are present at 6 months, amyloid plaques and neurofibrillary tangles starting at 6 years of age. Synaptic dysfunction occurs prior to the detection of plaques and tangles	[2, 3, 55]
Guinea pig	5–8 years	Amyloid-β deposition and Tau phosphorylation present in frontal cortex at 4 years of age	[7]

*Microcebus murinus* (gray mouse lemur) is a small nocturnal primate native to Madagascar that reaches the age of 8–14 years in captivity. Interestingly, at 5 years of age, 20 % of *M. murinus* show morphological signs of brain neurodegeneration [61] and cognitive decline [78, 79] that are similar to those of humans with AD [12, 31]. These signs include the accumulation of Aβ plaques in 15 % of the adult population, the formation of NFT [44, 63], and the loss of cholinergic neurons [72]. In *M. murinus* the gene responsible for the formation of Aβ plaques shows high sequence similarity with the human allele [12], and 92,7 % similarity with human ApoE4 allele [18] (Fig. 1). However, Aβ deposits and plaques have a different distribution in humans: they occur first in hippocampus, whereas in *M. murinus* they are first detected in other cortical areas [44]. These signs have allowed the population of old *M. murinus* to be classified into 4 groups: (i) animals presenting amyloid plaques without (5–10 %) or (ii) with a tauopathy (1 %); (iii) animals presenting tauopathy in the absence of amyloid plaques (1 %) and (iv) animals with no lesions (80–90 %). These data suggest that most of the mouse lemurs undergo a normal aging, whereas some of them show this age-associated pathology [63].

Another interesting species is *Cavia porcellus* (Guinea pig), a rodent native to the Andes Mountains. The aged guinea pig presents well-described, diffuse, amyloid-β deposits in the region of the hippocampus [7]. Interestingly its APP is highly homologous to the human protein (97 %) [10], and is processed in a similar way in vitro [9]. The APP695 variant is the most abundant form in the brain, whereas the longest isoforms are primarily expressed in peripheral organs [10]. High sequence similarity between human and guinea pig PSEN1 has also been documented, highlighting guinea pig as a good model for SAD [92]. However, the presence of senile plaques and neurofibrillary tangles and their correlation with cognitive and synaptic impairments has not yet been reported for the aged guinea pig brain.

The naked mole-rat (*Heterocephalus glaber*) (NMR) is a small rodent with a lifespan of over 30 years in captivity [16]. It presents early signs of neurodegeneration related to vitamin D deficiencies [17] and high levels of oxidative stress [1]. Moreover, appreciable levels of Aβ can be detected, and its sequence differs in only one amino acid with that of the human protein. Remarkably the mole rat brain acquires and tolerates high levels Aβ, but does not form plaques [38]. Moreover, tau phosphorylation levels rise during the early stages of life, resulting in an increase in molecular weight, reaching 88 kDa in the first year of life. This increase in phosphorylation declines at the end of development but occurs throughout life. Unlike other transgenic models such as 3xTgAD the phosphorylation in NMR is specifically localized in the axonal region but not in the somatodendritic compartment, where synaptic alterations could be generated [76]. Although phosphorylation is related to the loss of stability of microtubules, in NMR it is a tightly regulated kinase mechanism and it is essential for maintaining the dynamics of the cytoskeleton [76]. Interestingly, NMR tau protein is very similar to human tau (95 % similarity) and shows 100 % identity in the microtubule-binding domain, suggesting the inmportance of the location and regulation of the phosphorylation processes and not the sequence *per se.*

*Octodon degus* (degu) is a rodent native of South America, belonging to the Octodontidae family [96]. The *Octodon* genus includes three species: *O.degu*, *O. lunatus*, and *O. bridgesi* and is related to the Chinchilloidea and Cavioidea (e.g. *guinea  pig*) families [75]. During aging degus present intracellular and extracellular deposits of Aβ, intracellular accumulations of tau-protein, and strong astrocytic responses, suggesting that they represent a natural model for sporadic AD [55, 56]. More recently, Van Groen et al. [99] has shown that 6 year-old degus have Aβ and tau deposits in the hippocampus, and in the blood vessel walls. We have demonstrated that degu develops synaptic changes related to AD, which explains the early impairments in cognitive and neural plasticity observed before the appearance of fibrillar deposition [3]. We have also shown that the memory of degu declines during aging, correlated with an increase in the levels of soluble Aβ, in particular the Ab*56 oligomer. In a small number of cases, very old degus (7–9 years old) also seems to develop Aβ plaques, similar to what occurs in naked mole rats. Interesting, more recently we have shown that the retina (which is also part of the nervous system) of aged degus presents, as does the brain, the main hallmarks of AD [34].

The draft genome of the degu has recently been completed at the Broad Institute (Boston) and some important questions can now be addressed, such as the presence and homology of genes involved in the familial, or risk forms of human AD. Here we used this preliminary information to carry out bioinformatics analysis comparing genomic sequences of NTM.

## Methods

### Genomes

The species and protein sequence IDs used for this analysis were: **Aβ** (from APP sequence): *Homo sapiens* P05067.3, *Cavia porcellus* Q60495.2, *Octodon degus* XP_004627753.1, *Heterocephalus glaber* XP_004842285.1, *Rattus norvegicus* P08592.2; **ApoE:***Homo Sapiens* P02649.1, *Cavia porcellus* P23529.1, *Octodon degus* XP_004644379.1, *Heterocephalus glaber* XP_004910131.1, *Microcebus murinus* ENSMICP00000012801, *Rattus norvegicus* NP_001257613.1; **Tau:***Homo SapiensP10636.5*, *Cavia porcellus XP_003465958.1*, *Octodon degus XP_004630049*, *Heterocephalus glaber* EHB10652.1, *Microcebus murinus*, ENSMICP00000004446, *Rattus norvegicus P19332.3*; **Presenilin 1:***Homo Sapiens* P49768.1, *Cavia porcellus* XP_003472446.1, *Octodon degus* XP_004624901.1, *Heterocephalus glaber* XP_004837306.1, *Microcebus murinu*s CAA95930.1, *Rattus norvegicus* NP_062036.2.

## Results and discussion

### Phylogenetic trees

Multiple alignments followed by neighbor-joining analysis in order to generate the phylogenetic trees were performed using the Clustal X2 program as shown in Fig. 1.

AD is a neurodegenerative condition and is the main cause of dementia that affects over 24 millions of people worldwide [11]. AD occurs in two forms, the rare early-onset genetic or familial Alzheimer’s disease (FAD) which represents <1 % of diagnosed cases, and the frequent late-onset form or sporadic Alzheimer’s disease (SAD) [11]. Transgenes bearing mutations in genes linked to human FAD have been incorporated into mice to model the disease, and have provided valuable information about the neuropathology of the disease [36]. However, one difficulty associated with the use of this short-lived model is that aging is a main risk factor for AD. For this reason, it is imperative to search for long-lived animal models that can be used to study the neuropathology of AD.

Here we analyzed, for several natural models (naked mole, guinea pig, degus) as explained in the introduction, the sequences of proteins that have traditionally been associated with AD. These include ApoE, tau, Presenilins and Aβ (APP). In the resulting phylogenetic trees or phylograms, the length of the branches represents the amount of change in a sequence with respect to a common ancestor [8]. Our results show different relationships between species for some important markers of AD. Thus, we found that the degu is grouped with the mole rat and the guinea pig in all analyses; interestingly, the Aβ sequence for the guinea pig is identical to that of the human, because it includes a histidine at position 13 instead of the arginine, as occurs in other rodents. However, the most important change is likely to be H13R that is a critical residue for the aggregation of the Aβ [66]. Degu Aβ presents high homology with the human protein, differing in only one amino acid; this supports the hypothesis that the occurrence of amyloidogenic cascade is associated with the presence of Aβ oligomers [3] and amyloid deposits in older animals [55]. However, we also note that the mole rate shows 100 % homology (Fig. 1), presents high levels of Aβ [38], but does not show amyloid deposits. It will be important to determine if this rodent shows alteration in memory processes, which would establish it as a model of neurodegeneration and memory impairments in the absence of amyloid plaque. It will also be important to understand the markers present in the degu, as this rodent shows memory and synaptic impairments, and an accumulation of soluble amyloid oligomer but appears not to develop amyloid plaques.

Although our results for Presenilin 1 show a greater relatedness between the isoforms of human and, degu, guinea pig, and mole rat, compared to those of others rodents such the rat (Fig. 1c), there are more than 150 mutations in these proteins associated with Familial AD [26]; for this reason we need to perform more detailed analysis for each mutation to establish differences in the presence of potentially mutagenic variants in each of these species. Nevertheless, it should be noted that D257 and D385, two essential aminoacids of the catalytic site [101], are conserved in all species. In addition the aminoacid alanine 246, for which the substitution for a glutamate is associated with increased deposition of beta amyloid in transgenic models of AD [14], is also conserved in all species of this study. Interestingly, Sharman et al. reported that the S212Y mutation occurring in transmembrane domain 4 of PSEN1, and which was previously identified in a family with FAD [81], is conserved in guinea pig [92]. Additionally, a normal truncated PSEN2 isoform termed PS2V, which in humans has been implicated in AD [86], is also present in guinea pig but is absent in mice and rats [92], further confirming the close similarity between guinea pig and human presenilins.

In humans the ApoE gene has different isoforms that can be related to the occurrence of sporadic AD. These isoforms differ only in the variation of two amino acids and are designated as ApoE 2 (Cys112, Cys158), ApoE 3 (Cys112, Arg158), and ApoE4 (Arg112, Arg158). Within these isoforms ApoE 4 has been described as the major risk factor in sporadic late onset Alzheimer’s disease (LOAD) [67], where homozygosity for the E4 allele may be sufficient to develop AD at the age of 80 years [24]. This allele has a single amino acid change with respect to the E3 allele (C112R) [24]. ApoE is fundamental for the regulation of clearance and deposition of Aβ in the brain, which may explain its association with sporadic cases of AD [67]. Studies in HEK-293 cells demonstrate that the binding between ApoE and Aβ is less effective for ApoE4 than ApoE3 [62]. On the other hand ApoE can disrupt the clearance of Aβ from brain mouse in an isoform specific manner [30]. These findings suggest that the differences in the amino acids between the three isoforms of ApoE can fundamentally affect its role in the regulation of Aβ.

Surprisingly, we found that degu have the arginine substitution present in the ApoE4 pathogenic human allele. In addition, our results show that, in the sequence of human of ApoE 3, the amino acid at position 112 is a cysteine, unlike degu and other rodents, which have an arginine at this position as is found in the human ApoE4 form. These data would suggest that this protein could potentially be pathological for the rodents. However all rodents have this amino acid, not only those that show sporadic AD such as the degu. Thus, the functional consequences of these substitutions await further sequence and protein structure analyses.

One possible explanation for this anomaly arises from the fact that ApoE has a region that is essential for binding lipids. In humans, this region has a glutamate at position 255 that is critical for the structure of the “toxic” E4 allele because it generates an ionic interaction with Arg61 that changes the position of the lipid binding region and the protein structure. On the other hand, the presence of Cys112 in the E3 allele leads to an interaction between this amino acid and Arg61, causing Glu255 and Arg61 not to form the interaction that occurs in E4 [69]. Studies in cell culture show that the substitution of an Arg at position 61 for a Thr generates a disruption in the interaction with Glu255, generating an E3 like structure [103]. In the case of rodents such as degu this amino acid is a Thr so an interaction with the lipid binding region would not possible despite the presence of Arg112. Thus, the presence of Arg112 may not be a “toxic” substitution in rodents. However, it is clear that amino acids Thr61, Arg112, and Glu255 are important for maintaining the function of ApoE. More research is needed to determine the presence of polymorphisms in these and other amino acids that can account for potential pathogenic substitutions that are present in models of sporadic AD such as degu.

On the other hand, it has been speculated with the possibility of certain “protective” polymorphisms in the case of human ApoE, since that inheritance of ApoE2 has been associated with a decrease in the AD risk [39]. This allele has a lower affinity to LDL compare to other alleles [42], leading to a decrease in their clearance resulting in an increase in plasma, cerebrospinal fluid and brain levels [70]. That increase in ApoE2 availability in the brain could explain the greater Aβ clearance observed in human and mice models. [5, 20]. In the E2 allele, Cys158 causes Asp154 to now interact with Arg150, thus modifying the LDL binding domain [33]. By contrast, in the E3 allele the amino acid Arg158 forms a salt bridge interaction with Asp154, which does not affect the LDL binding domain (amino acids 134–150).

In degu, the amino acids at these positions are those present in the E3 allele (Arg150, Asp 154, Arg158). Thus, it will also be necessary to determine the presence of different protective polymorphisms in this rodent. Consistent with this, brain levels of ApoE are altered (in the frontal cortex and hippocampus) depending on ApoE genotype, where animals homozygous for the E4 allele have lower protein levels than those with the E3 allele, which in turn are lower than those with the E2 allele [80]. Genetic and crystallographic research is needed to understand the consequences of each of the various polymorphisms on ApoE protein structure and function. It will also be important to determine how hetero- and homozygosity for the different ApoE alleles might affect the onset of sporadic of AD in the degu.

Interestingly, a recent report by Deacon et al. [29] shows that poor-burrower degus have high levels of Aβ_1–42_, APOE, and APP, cytokine TNF-α and oxidative stress marker NFE2L2 compared to good-burrowers degus. This result, although preliminary, suggest that degus present increased inflammation during aging and AD like diseases, adding a new group of protein targets to be considered in this type of bioinformatics analysis.

Regarding the tau protein, we found that for all rodents its sequences are grouped in a branch of the tree that is separate from that for lemurs and humans (Fig. 1), which show the highest similarity [76]. Since there is no evidence for a link between polymorphisms in tau and risk of AD, and the SNPs identified in the MAPT gene do not influence the risk of AD [59], we cannot extrapolate much from these data. However, the regulation of MAPT transcriptional splicing is known to be critical for normal tau function [48]. It has also been shown that an imbalance in the ratio of tau isoforms with 3 (3R) vs. 4 (4R) repeats, which are derived from the alternative splicing of Exon10 of the human MAPT, is associated with AD and Tauopathies [19]. In this regard, guinea pig show both 3R and 4R tau isoforms and, interestingly, under cholesterol intake, guinea pig shows an increase in 3R isoforms suggesting a relationship between AD risks factors and AD in guinea pig [92]. It would be interesting to determine if the 3R/4R ratio also is disturbed in the other NTM’s.

Overall, our analyses reveal that the sequences of genes associated with AD risk are more similar to human in NTM than in rat or mouse, explaining why the biomarkers encoded by these genes appear during aging in NTM. The fact that the temporary or regional expression of these markers differ between the different models discussed here provide an opportunity to explore different factors that can accelerate the progress of AD-related pathological events. The understanding of these processes also provides a unique opportunity to explore new drugs or therapeutic strategies against this devastating disease.
